# Which Frail Older Patients Use Online Health Communities and Why? A Mixed Methods Process Evaluation of Use of the Health and Welfare Portal

**DOI:** 10.2196/jmir.3609

**Published:** 2014-12-17

**Authors:** Peter Makai, Marieke Perry, Sarah HM Robben, Henk Schers, Maud Heinen, Marcel GM Olde Rikkert, René JF Melis

**Affiliations:** ^1^Radboud University Medical CenterRadboud Institute for Health SciencesNijmegenNetherlands; ^2^Jeroen Bosch HospitalDepartment of Internal Medicine's-HertogenboschNetherlands

**Keywords:** eHealth, frail older people, care-coordination, chronic care

## Abstract

**Background:**

Frail older people often receive fragmented care from multiple providers. According to the literature, there is an urgent need for coordination of care. Online and eHealth tools are increasingly used to improve coordination. However, there are significant barriers to their implementation in frail older people.

**Objective:**

Our aim was to (1) evaluate differences in use of a personal online health community (POHC) for frail older people in relation to personal characteristics, and (2) explore barriers and facilitators for use as experienced by older people and their informal caregivers, using the case of the Health and Welfare Information Portal (ZWIP).

**Methods:**

This is a mixed methods study. For the quantitative analysis, we used POHC usage information (2 years follow-up) and baseline characteristics of frail older people. For the qualitative analysis, we used semistructured interviews with older people and their informal caregivers. Participants were recruited from 11 family practices in the east of the Netherlands and frail older people over 70 years. The ZWIP intervention is a personal online health community for frail older people, their informal caregivers, and their providers. ZWIP was developed at the Geriatrics Department of Radboud University Medical Center. We collected data on POHC use for 2 years as well as relevant patient characteristics. Interview topics were description of use, reasons for use and non-use, and user profiles.

**Results:**

Of 622 frail patients in the intervention group, 290 were connected to ZWIP; 79 used ZWIP regularly (at least monthly). Main predictors for use were having an informal caregiver, having problems with activities of daily living, and having a large number of providers. Family practice level predictors were being located in a village, and whether the family practitioners had previously used electronic consultation and cared for a large percentage of frail older people. From 23 interviews, main reasons for use were perceiving ZWIP to be a good, quick, and easy way of communicating with providers and the presence of active health problems. Important reasons for non-use were lack of computer skills and preferring traditional means of consultation.

**Conclusions:**

Only 27.2% (79/290) of frail older enrolled in the POHC intervention used the POHC frequently. For implementation of personal online health communities, older people with active health problems and a sizable number of health care providers should be targeted, and the informal caregiver, if present, should be involved in the implementation process.

**Trial Registration:**

International Standard Randomized Controlled Trial Number (ISRCTN): 11165483; http://www.controlled-trials.com/isrctn/pf/11165483 (Archived by WebCite at http://www.webcitation.org/6U3fZovoU).

## Introduction

### Potential Benefits of eHealth for Frail Older People

Frail older people have a large number of health deficits and receive fragmented care, often resulting in nursing home admissions and hospitalization [1]. Frail older people also have multiple health care providers. The collaboration between these providers is not always optimal, and they are often unaware of the fact that they care for the same patient. This reveals insufficient coordination in the care for frail older people [2]. There are several barriers to providing coordinated care to frail older people in a cross-institutional setting: (1) the physical barriers of working in different institutions located in different geographical locations, (2) not having a timely overview of all professionals involved in the care of the patient, (3) information that colleagues have on the patient, (4) how they treat the patient, and (5) that multidisciplinary consultation can take place only if all professionals are available at the same time. The required coordination of care for frail older people may be facilitated by the implementation of eHealth [3,4].

Personal online health communities (POHCs) seem particularly suited to improving the coordination of care for frail older people with multiple providers. Such communities allow a patient, and/or professionals caring for a specific patient, to interact in online networks. In general, online health communities are online platforms that unite providers and/or patients with a shared goal or similar interest [5]; in the case of POHCs, that is the care for an individual. POHCs enable communication between people who would not have met each other otherwise [5]. They also provide an alternative to face-to-face consultations. POHCs facilitate communication by organizing a network of providers around a patient [5]. They are a relatively new development in eHealth [6], and so information on the determinants of usage is limited. We developed the Health and Welfare Information Portal (in Dutch: Zorg- en WelzijnsInfoPortaal or ZWIP), a POHC aimed at reducing fragmentation in care in frail older people. ZWIP targets individual patients, their informal caregivers, and their health care providers, to learn about the feasibility and effects of POHCs in the care for frail older people. In an earlier study, we showed that ZWIP had not yet yielded any benefits [7], but there were indications that usage was heterogeneous across users and, in general, limited. Usage is therefore the focus of this study.

### Determinants of Using Personal Online Health Communities in Older People

Despite the potential benefits of POHCs, implementing eHealth is difficult, especially in frail elderly populations [3]. Implementation difficulties in this group arise because frail older people have decreased physical and cognitive function leading to a loss of autonomy. Older people in general have a lower level of computer literacy than younger populations, which has to be considered when developing and implementing eHealth interventions for this group [8]. Furthermore, older patients may not be able to use complex and multifunctional eHealth interventions, which also leads to low levels of usage.

At present, little is known about facilitators of eHealth usage in general and POHCs in particular in frail older people and how the barriers presented can be overcome [3]. Previous studies focused on implementation barriers during the introduction of eHealth interventions in other groups, such as health care providers [9-14] and in younger patients with chronic illnesses [15-17]. Knowledge about such determinants of usage in frail older people would allow identification of older people who are likely to use and possibly benefit from eHealth interventions.

### Aim

The aim of this study was to investigate the main determinants of ZWIP usage in frail older people. First, we describe ZWIP use and its users’ characteristics. Second, we investigate the predictors (barriers and facilitators) of ZWIP usage in frail older people to identify successful users.

## Methods

### Intervention

ZWIP is a personal online health community for multidisciplinary communication and information exchange for frail older people and their informal caregivers. The development of the intervention has been described elsewhere [2]. Briefly, ZWIP is a secure digital environment, aiming to improve collaboration within the care network around frail older people. Within the ZWIP network, frail older people (or on their request, their informal caregiver) take the lead. Patients and their informal caregivers give permission to providers to join their network. For all members this creates an overview of the providers involved in their care. ZWIP contains a message system similar to email, where patients can exchange messages with these providers. Most messages are visible to all members of a patient’s network, with the exception of private messages. Therefore, all relevant professionals and informal caregivers are kept up to date on care-related developments. Based on the family practice’s information system and frailty identification through Easycare-TOS (see below), ZWIP contains current medical and social care data on frail older people, which is shared within a patient’s network. ZWIP also gives the opportunity to register individual patient’s care-related goals and action plans. Goals and action plans are determined by the patients in consultation with the professionals. Patients may also receive tailored health information via ZWIP. (See Multimedia Appendix 1 for a video of a patient using ZWIP.)

### Centers, Participants, and Recruitment

ZWIP was implemented in 11 family practices in the east of the Netherlands. The participating practices started by screening all of their patients aged 70 years and older in alphabetical order using the Easycare-TOS (Two step Older persons Screening) instrument. Easycare-TOS is a validated, two-step measure to identify frail older people in the community as a target population for integrated geriatric care [18]. Easycare-TOS is based on the EASYcare-system, a focused geriatric assessment by a primary care nurse [19,20]. Initially, the family practitioner completed a questionnaire in Step 1 and judged patients as frail, frailty uncertain, or not frail based on readily available information. If patients were assessed as being frail or family practitioners were uncertain, a second assessment step (Step 2) was carried out by specialized nurses to confirm or exclude frailty. All 622 patients who were considered frail based on the screening were invited to participate in ZWIP, of which 290 (46.6%) consented.

### Design

In complex interventions, which are usually difficult to describe and replicate, qualitative research can help clarify the processes of implementation and change. Therefore, we evaluated ZWIP usage with a mixed methods study. The local ethics committee (Committee on Research Involving Human Subjects Region Arnhem-Nijmegen) stated that no formal approval was required due to the non-invasive and non-experimental nature of the study [21,22].

### Quantitative Data Collection

Quantitative data were collected in the intervention arm of an effectiveness trial [7], and measurements were performed by trained nurses in the patients’ homes using a face-to-face questionnaire. We used baseline data for demographics, physical, mental and social functioning, as well as coordination of care and care use. We included patient characteristics observed by the family practitioner or obtained during the Easycare-TOS assessment. Individual baseline characteristics used were the Barthel index of Activities of Daily Living (ADL) and the Lawton scale of Instrumental Activities of Daily Living (IADL), the Short Form-36 Health Survey (SF-36) (social activity limitations and mental health) [23-26], as well as education, age, and sex. Family practice level characteristics were practice location, family practitioner having used electronic consultation previously, and percentage of frail older people in the practice. Furthermore, we used data from the ZWIP application’s logging files. In this model, ZWIP usage was operationalized as monthly total page views by the patients or their informal caregivers. ZWIP use was followed up to 2 years. We operationalized ZWIP usage as a combined measure of total page views by the patients or their informal caregivers. To capture all forms of usage, we counted passive usage (simply logging in without interaction with the providers, viewing health promotion) and active usage by the older people or their informal caregivers (actual communication with providers, adapting goals).

### Quantitative Data Analysis

Baseline characteristics of ZWIP participants and non-participants were compared using *t* tests and chi-square tests. We classified different groups of users according to usage of ZWIP, using hierarchical cluster analysis [27], creating groups from observations based on similarities of their features. ZWIP users were clustered according to the degree of use of the various ZWIP features (communication, goals, health promotion, etc) [27]. Clusters were created using the Ward method [28], and the number of separate groups were determined using the pseudo *t* ^2^ and the pseudo *F* ^2^ [27,29].

Furthermore, we investigated the relationship between usage and baseline characteristics at the patient and family practice levels. We used a hierarchical linear growth model [30] to account for potential variation in use across time (months since connection to ZWIP) within a patient, between patients with various characteristics, and between family practices. All analyses were performed using SAS 9.2.

### Qualitative Data Collection

Participants were patients and their informal caregivers who participated in ZWIP and were selected using purposive sampling [31]. To include a wide variety of users and levels of use during sampling, we allowed for the following patient characteristics: sex, age, family practice, whether a patient or an informal caregiver managed the patient’s ZWIP account, network size, and how much they used ZWIP (ZWIP usage). If any patient declined to participate, a patient with comparable characteristics was chosen instead. The final sample size was dictated by data saturation [32].

Three research assistants, who were not otherwise involved in the evaluation of ZWIP, approached the selected patients or their informal caregivers and conducted individual semistructured interviews at the patients’ homes. The interviewers used a topic list based on relevant themes derived from the literature [33], which evolved when relevant new topics came up. Initial topics were description of use, reasons for use/non-use, and user profiles. New topics were discussed in the research group and pursued if relevant to the aim of this study. All interviews were audiotaped and transcribed verbatim. Data collection continued until saturation was reached (eg, when no new themes emerged from the interviews).

### Qualitative Data Analysis

To analyze the transcripts, 3 researchers (PM, SR, and MP) used directed content analysis [34] and deconstructed the interviews independently, using the principles of iterative comparison. In discussion meetings, PM, SR, and MP reached agreement on codes, combined codes to generate and adjust categories (axial coding), merged categories to compose and refine themes (selective coding), and discussed whether saturation had been reached. We used Atlas-ti 5.2 software to help in data coding and retrieval.

### Integration of Quantitative and Qualitative Data

Quantitative data were used to identify qualitative respondents. We based the user profiles on the clusters identified during cluster analysis, thus making the link between qualitative and quantitative data. Qualitative and quantitative data on facilitators for use were presented separately and integrated by triangulation [35] in the discussion. Similar weight was given to the two types of analyses.

## Results

### Participants

A total of 290 vulnerable older patients (46.6% of all 622 eligible patients) completed baseline measurement and participated in ZWIP. The other 332 refused participation. ZWIP participants were similar to non-users in terms of demographic criteria, functional measures such as ADL and mental health measures, number of illnesses, and the types of illnesses present with the exception of dementia. Furthermore, they were similar in terms of a number of process indicators such as patient experience with care measures, and family practitioner reported process measures, with one exception: patients using ZWIP were more likely to have more providers involved in their care (Table 1).

In total, 23 patients and informal caregivers participated in the interviews. Patient age varied between 74 and 90 years, and they came from a wide range of practices (see Multimedia Appendix 2). Additionally, as a result of purposive sampling, interviewed patients varied substantially in terms of messages sent and received and if they had an informal caregiver and the type of informal caregivers (spouse or child).

### Usage

The most frequently used ZWIP function was messaging with an average of 12 messages per patient during the 2-year follow-up period (Table 2). Additionally, 47.9% (139/290) of patients used the goal-setting function and 13.1% (38/290) of older people modified or evaluated the goals during ZWIP usage. Furthermore, 33.1% (96/290) of patients had defined concrete care-related activities to reach their goals; in the case of 9.0% (26/290) of patients, these activities were modified or actively evaluated in ZWIP during the period of ZWIP usage.

**Figure 1:**
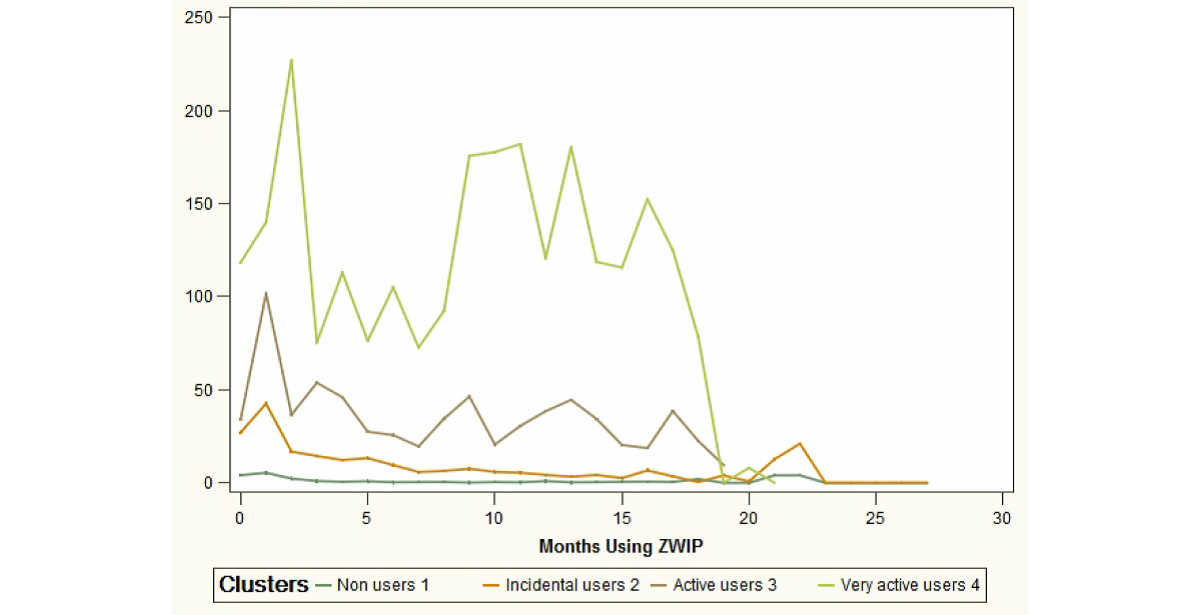
Average page views per month of usage for the 4 identified usage clusters.

Hierarchical cluster analysis identified four main groups of users: Non-active users (using ZWIP only a few times within 2 years), Regular users (once a month), Active users (once a week), and a small group of Very active users (daily users). From quantitative demographic data and qualitative data, average user profiles per cluster were constructed (Textbox 1). As can be seen in Figure 1, after an enthusiastic start the number of page views decreased to remain relatively stable in all groups a few months after the start, except for the very active users (Figure 1).

In addition, qualitative data further characterized the variations in use seen in the quantitative data trends. Qualitative data confirmed that patients and informal caregivers made highly variable use of ZWIP: “I check ZWIP every day” [Respondent 21, Patient], “I probably checked ZWIP 2 or 3 times” [Respondent 6, Informal caregiver], and “At the start I checked ZWIP once or twice a week. Later, it decreased” [Respondent 16, Patient].

The number of providers that patients had contact with was also highly variable. Whereas some patients never logged in, only added informal caregivers to their network, or waited for others to communicate, other patients used ZWIP intensively to communicate in their network: “Only the family practitioner and district nurse, with the other health care providers I did not have any contact” [Respondent 14, Informal caregiver].

Other patients mentioned that providers did not respond to the invitations for their networks and that they got little response to their messages from providers. Interviewed ZWIP users and informal caregivers used only the communication platform: “I only used the communication tool” [Respondent 10, Informal caregiver].

**Table 1:** Comparison of frail older patients who used and did not use ZWIP.

Characteristics		Total	Used ZWIP	Did not use ZWIP	*P* value (2-sided)
Female, n (%)		400	180 (63.2)	220 (66.7)	.36
**Education, n (%)**					.25
	≤Primary education	136	56 (21.7)	80 (28.7)	
	Secondary education	380	192 (74.4)	188 (67.4)	
	University / tertiary education	21	10 (3.9)	11 (3.9)	
**Marital status, n (%)**					.43
	Married	253	128 (44.4)	125 (38.1)	
	Divorced	29	11 (3.8)	18 (5.5)	
	Widow / widower / partner deceased	290	132 (45.8)	158 (48.2)	
	Unmarried	38	15 (5.2)	23 (7)	
	Long-term cohabitation, unmarried	6	2 (0.7)	4 (1.2)	
Living independently, n (%)		314	134 (48.2)	180 (55.4)	.08
Informal caregiver, n (%)		484	242 (84.0)	242 (74.0)	.003**
Informal caregiver lives together with the frail older person, n (%)		242	132 (56.2)	110(46.2)	.03*
Benefit expected from more coordinated care according to family practitioner, n (%)		228	102 (39.5)	126 (43.9)	.30
**Number of other care providers than the family practitioner involved, n (%)**					.007**
	No other care providers	75	22 (7.6)	53 (16)	
	1-3 other care provider	407	200 (69.4)	207 (62.3)	
	More than 3 other care providers	138	66 (22.9)	72 (21.7)	
Age in yrs, mean (SD)		615	82.13 (5.77)	82.07 (5.72)	.89
Frailty index (scale 0-1, higher is worse), mean (SD)		616	0.29 (0.08)	0.30 (0.08)	.16
Barthel index (0-20, 20 completely independent), mean (SD)		616	16.01 (3.91)	15.99 (3.66)	.95
**Morbidity, n (%)**					
	Diabetes	146	68 (23.9)	78 (23.6)	.95
	Stroke	91	38 (13.3)	53 (16.1)	.34
	Heart failure	55	26 (9.1)	29 (8.8)	.88
	Cancer	76	39 (13.7)	37 (11.2)	.35
	COPD	100	44 (15.4)	56 (17)	.61
	Urinary incontinence	144	62 (21.8)	82 (24.8)	.37
	Osteoarthritis	2	1 (0.4)	1 (0.3)	.92
	Osteoporosis	39	16 (5.6)	23 (7)	.49
	Hip fracture	17	6 (2.1)	11 (3.3)	.35
	Fractures other than hip	12	7 (2.5)	5 (1.5)	.40
	Dizziness with falling	15	7 (2.5)	8 (2.4)	.98
	Benign prostate enlargement	16	10 ( 3.5)	6 (1.8)	.19
	Depression	10	2 ( 0.7)	8 (2.4)	.09
	Anxiety / panic disorder	16	11 (3.9)	5 (1.5)	.07
	Dementia	25	19 (6.7)	6 (1.8)	.002**
	Hearing problems	197	98 (34.4)	99 (30)	.25
	Vision problems	133	60 (21.1)	73 (22.1)	.75
Multimorbidity, mean (SD)		615	1.76 (1.42)	1.80 (1.34)	.68
Katz IADL (0-8, 8 completely limited), mean (SD)		616	5.06 (2.92)	5.46 (2.77)	.08
SF-36 Social activity limitations (1-5,with 5 no limitations), mean (SD)		616	1.58 (1.39)	1.57 (1.40)	.90
SF-36 mental health (1-100, 100 perfect mental health), mean (SD)		604	63.31 (13.11)	63.47 (13.12)	.90
Patient experience with being informed (1-5, with 5 optimal), mean (SD)		512	4.79 (0.67)	4.74 (0.78)	.43
Patient experience with coordination of care (1-5, with 5 optimal), mean (SD)		327	4.65 (0.88)	4.72 (0.82)	.45
Patient experience with co-decision making (1-5, with 5 optimal), mean (SD)		486	4.86 (0.54)	4.84 (0.60)	.71
Patient preferences for influence (1-5, with 5 optimal), mean (SD)		551	3.46 (1.13)	3.50 (1.10)	.68
Patient knowledge of providers (health and social) (1-5, with 5 optimal), mean (SD)		587	3.56 (0.65)	3.60 (0.69)	.38
Patient experience with self-management (1-5, with 5 optimal), mean (SD)		497	4.86 (0.52)	4.91 (0.45)	.20
Number of emergency family practitioner visits during past 12 months, mean (SD)		520	0.74 (2.08)	0.61 (1.22)	.38
Unplanned hospitalization during past 12 months, mean (SD)		535	3.52 (7.18)	3.85 (12.37)	.70
Degree of certainty about treatment (according to family practitioner) (0-10, with 10 complete certainty), mean (SD)		621	6.13 (2.42)	6.44 (2.38)	.10
Agreement between providers involved with the patient (according to family practitioner) coordination of care around patient (0-10, with 10 lot of experience), mean (SD)		621	5.68 (2.58)	6.02 (2.38)	.09

*P<.05.

**P<.01.

**Table 2:** Usage of the different functionalities of ZWIP, as used in the cluster analysis for the four different groups of users.

	Total group	Non-users	Regular users	Active users	Very active users
People using ZWIP, n (%)	290	211 (72.8)	67 (23.1)	9 (3.1)	3 (1.0)
Older people logged in at least once, n (%)	220	141 (64.1)	67 (30.5)	9 (4.1)	3 (1.4)
Number of providers in network per older person at baseline, mean (SD)	4.44 (2.77)	3.80 (2.20)	5.76 (3.28)	7.56 (3.40)	10.67 (0.58)
Page views per patient during 2 yrs, mean (SD)	102.64 (273.16)	19.32 (22.71)	188.22 (78.74)	659.33 (174.88)	2381.33 (490.02)
Messages sent in a network during 2 yrs, mean (SD)	11.54 (35.26)	2.47 (4.44)	19.94 (21.02)	70.89 (49.08)	284.33 (122.10)
Other ZWIP functions (health promotion usage, setting and modifying goals, actions to reach the goals) used during 2 yrs, mean (SD)	7.22 (6.62)	5.53 (3.23)	10.07 (7.86)	19.33 (12.83)	26.00 (27.78)

**Example user profiles per group.**

Non-active user

A man in his 70s, suffering from heart failure and stroke. His daughter already predicted the limited use of ZWIP by her father, because his computer skills were poor. She lived very close to her father but also hardly used ZWIP for her father. Even though ZWIP seemed appealing to her, she logged in only three times. She agreed to participate in ZWIP in view of the future digitalization of society. The daughter found ZWIP redundant. The family practice and the pharmacy were across the street from where she lived, which facilitates face-to-face instead of digital consultation.

 

Regular user

A woman in her 90s, suffering from dementia, heart failure, and deafness. She was living together with her son, who managed her ZWIP. The family practitioner and the practice nurse participated in the patient’s ZWIP network. Her son liked to occasionally use the ZWIP to ask questions regarding his mother’s treatment and background information on her medical conditions. Her son also rescheduled his mother’s appointments through the ZWIP. When his mother became more apractic due to dementia, the occupational therapist was invited to join ZWIP. Her son appreciated ZWIP for its user friendliness, the easy small-scale communication, and the complete overview of his mother’s conditions and medication use.

 

Active user

A woman in her 80s, suffering from coronary heart disease. Her deceased husband was a health care provider. She managed ZWIP herself. Generally, she found it difficult to reach the family practice. What she appreciated most from ZWIP was that the family practitioner was easily accessible for health- or medication-related questions. That made her feel safe during the time of her myocardial infarction and it prevented, in her view, unnecessary face-to-face consultations in the family practice. She also kept her family practitioner informed of her condition after visits to the cardiologist. The family practitioner was her only active contact in ZWIP and that was all she needed. At the time of the interview, she was doing relatively well, so she hardly used ZWIP in that period.

 

Very active user

A man in his 80s, suffering from COPD and heart failure. He was highly educated and had good computer skills. He used ZWIP daily. He strongly appreciated the two main components of ZWIP: the messaging system and especially the goal-setting function. His family practitioner, cardiologist, and pulmonary nurse participated in his ZWIP. He reported that the frequency of the home visits has decreased and the physically exhausting face-to-face consultations with his health care providers had become rarer.

### Perceptions of ZWIP Usage

Perceptions of ZWIP usage were rather positive, illustrated by the quotes below. Some patients and informal caregivers perceived that communication was quicker than by telephone and that ZWIP was a good way to co-decide on their own health and welfare issues with the provider: “It is fast, multidisciplinary and you are in control of your own care. Very pleasant!” [Respondent 22, Patient] and “You send a message, and when it is convenient for them they answer. I think it is easier for them, also for the family practitioner” [Respondent 20, Informal caregiver].

Exchange of information and access to providers was perceived to be better using ZWIP: “I think it (ZWIP) causes unnecessary appointments to be avoided and at the same time it provides a safe connection to the family practitioner” [Respondent 17, Patient].

Informal caregivers said the ZWIP gave them a complete overview of the patient’s situation: “You can paint a more complete picture than on the phone.” [Respondent 10, Informal caregiver] and “My parents needed more care. It seemed like a good idea to get a good overview and simplify communication” [Respondent 10, Informal caregiver].

Other participants were disappointed by the frequency of communication through ZWIP: “Well, we expected that there would be more information exchange...” [Respondent 20, Informal caregiver].

### Predictors for Use: Barriers and Facilitators

In quantitative user analysis, several determinants of use were identified (Table 3). When a person had larger networks involved in their care according to the family physician, this led to more page views. Having an informal caregiver also contributed positively to ZWIP usage. Also, patients with a higher Barthel index (better ADL) at baseline showed fewer page views compared to the older people with lower Barthel index (worse ADL) at baseline. All patients slightly decreased in their use of ZWIP over time. On a family practice level, having a higher percentage of frail older people in the practice, previously having used electronic consultation methods, and being located in a village (versus a city) led to more active ZWIP usage.

Also, a major theme addressed in the interviews was facilitators of use. According to the interviewees, a successful older ZWIP user was interested in communicating about health and welfare status and was pro-actively sending messages to providers using ZWIP: “I like to be in control of my own care. I can handle different opinions. And I have several health problems, on which I have questions” [Respondent 22, Patient, on why ZWIP was successful in his case].)

Importantly, these patients had current, chronic health problems for which they used ZWIP. Patients using ZWIP should have computer skills and should have no cognitive problems, or should have an adequate informal caregiver who can use ZWIP on their behalf: “I can handle it (computer) very well. My mother cannot, so I support her” [Respondent 18, Informal caregiver]. ZWIP was considered a good, quick, and easy communication tool: “I can easily communicate without picking up the phone for everything” [Respondent 10, Informal caregiver].

Informal caregivers, especially children who were living far away, mentioned that ZWIP made it easy for them to be involved in the care for their vulnerable parent. “It is a triangle, it is about my husband, but it could also support me” [Respondent 19, Informal caregiver] and “Before, you had to wait until the family practitioner came. Now we used ZWIP and the family practitioner was already informed when he came” [Respondent 3, Informal caregiver].

An important advantage compared to telephone and face-to-face communication was that in ZWIP it is possible to communicate at a time that is convenient for patients and providers: “A small example is that I do not have to take my mother to the family practitioner for everything. I can just ask a question” [Respondent 18, Informal caregiver].

Some patients mentioned that they used ZWIP because it gave them a sense of safety, being able to communicate with their health care providers and to ask questions in ZWIP: “It is good that I can alarm providers about my father’s situation, rather than them hearing only his story” [Respondent 18, Informal caregiver]. One patient said that communication in ZWIP could replace a consultation, which was convenient for him because his medical condition made it difficult for him to visit his physicians: “Before, you had to wait until the family practitioner came. Now we used ZWIP and the family practitioner was already informed when he came” [Respondent 3, Informal caregiver].

Another major theme in the interviews was barriers for use. Patients or informal caregivers who did not or hardly used ZWIP often stated that there was no need for ZWIP, since they or the older people they cared for were healthy: “There was no reason for use. My father is actually still very healthy” [Respondent 15, Informal caregiver] and “Why should I communicate if I do not have any questions?” [Respondent 23, Patient].

Some older people simply had problems logging in but never asked for help, or they reported that support was not sufficient: “I thought it was all very complicated…I could not handle it” [Respondent 9, Informal caregiver] and “A computer is a little impossible for an older person” [Respondent 15, Informal caregiver].

However, patients did expect an increased usage of such interventions in the future when computer skills are more common among older patients. Other patients did not like the fact that ZWIP was a computer app and were not interested in using it. Some patients feared that the ZWIP would replace face-to-face contact with their health care providers and preferred to phone or visit the family practice: “You do not see your family practitioner anymore. That is a disadvantage… When contact is only digital, I think care becomes rather meager” [Respondent 16, Patient].

Other patients and informal caregivers would have wanted to use ZWIP but complained that they got no response or only a limited response from health care providers. This was particularly problematic if it was their family practitioner not responding. “We experienced slow reactions from health care providers…So we could not use it for communication” [Respondent 20, Informal caregiver].

Some of these professionals did not even accept the invitation to participate in the patient’s network. Several patients said that they assumed that their family practitioners were too busy to communicate in ZWIP and so did not try further: “I found it cumbersome. And because I thought: they are never going to make time for that. They are already so busy” [Respondent 6, Informal caregiver].

**Table 3:** Association of potential patient-level and family practice/practitioner-level predictors with the number of total page views at baseline and across 2 years of follow-up using hierarchical linear models.

Baseline determinants (fixed effects)		Time-determinant determinants (linear rate of change)	Estimate	95% CI	*P* value
Intercept			-3.16	-19.41 to 13.09	.70
**Patient-level determinants**					
	Network size (number of professionals at baseline)		2.75	1.83-3.67	<.001***
	Education		1.39	-0.20 to 2.98	.09
	Barthel index of ADL (0-20)		-0.94	-1.67 to -0.21	.012*
	SF-36 mental health (0-100)		-0.15	-0.31 to 0.01	.07
	Age at baseline		0.08	-0.29 to 0.45	.69
	Multimorbidity		-0.75	-2.30 to 0.80	.35
	Informal caregiver		6.67	0.71-12.63	.03*
	Sex (female)		-3.32	-8.14 to 1.50	.18
	**Account closed**		5.45	-10.76 to 21.66	.51
		Time (months from baseline)	-0.03	-1.48 to 1.42	.96
		time*network size (number of professionals at baseline)	-0.18	-0.26 to -0.10	<.001***
		time*education	-0.10	-0.28 to 0.08	.25
		time*barthel index at baseline	0.09	0.01-0.17	.02*
		time* SF-36 mental health at baseline	0.02	0.00-0.04	.06
		time*age at baseline	-0.05	-0.09 to -0.01	.03*
		time*multimorbidity at baseline	0.05	-0.13 to 0.23	.53
		time*informal caregiver at baseline	-0.55	-1.20 to 0.10	.10
		time*sex	0.43	-0.08 to 0.94	.11
		time*account closed	-0.20	-1.32 to 0.92	.72
**Family practice-level determinants**					
	Village practice		24.25	15.06-33.44	<.001***
	Electronic consultation		10.92	1.00-20.84	.03*
	**Percent frail**		0.95	0.48-1.42	<.001***
		time*village	-0.16	-1.14-0.82	.75
		time*electronic consultation	-0.33	-1.39 to 0.73	.54
		time*percent frail	0.00	-0.06 to 0.06	.92

*P<.05.

**P<.01.

***P<.001.

## Discussion

### Principal Findings

Active use of ZWIP as an innovative POHC, specifically developed for frail older people, was limited, despite a small group of very active users. However, in active networks ZWIP was highly appreciated. ZWIP was mainly used for communication. The combination of quantitative and qualitative data revealed a distinct profile of an active ZWIP user: a patient or informal caregiver interested in communicating about their own or their relative’s health and welfare status and sufficiently pro-active to actually stimulate communication in ZWIP. Importantly, these patients had current, chronic health problems that they used ZWIP for and had a lower functional performance (low Barthel scores). Multiple health care providers in a patient’s network (reflected in network size) was also associated with increased ZWIP use.

### Characteristics of Likely Users

The current study contributes to identifying characteristics of the older people who are likely to use POHCs—either on their own or with additional support—and which older people are unlikely to use a POHC. It seems that additional targeting criteria are useful for the successful implementation of POHCs for frail older people. Whereas some general characteristics may be applicable for all potential users of eHealth interventions, others seem specific for this population of frail older people. In line with previous research [36], impaired health status (reflected in ADL functioning and indirectly in network size) was an important predictor for using a website in older people. This indicates that a minimal level of care need was required. So there needs to be a current reason for intensive contact with health care providers for a POHC to be used. However, other characteristics identified by previous research [37] such as education were not significantly related to ZWIP use. This may be due to the generally low level of education of the older people in the sample compared to younger populations. Additionally, patient age and gender seem to be a common predictor of usage [33]; however, in this study neither was significant. This may be due to the low level of ZWIP’s use. At the same time, the effect of age is partially captured by the health status variable, as shown by a small and significant negative interaction between age and continued usage (analysis on request). Further research is needed to investigate how age influences usage beyond health status.

Two additional characteristics of successful implementation are worth mentioning. On a family practice level, this study shows that a high percentage of frail older people in the family practice’s patient population led to more ZWIP use. This finding suggests that context effects are also important for usage. Additionally, having an informal caregiver significantly contributed to ZWIP’s increased usage, which may indicate that informal caregivers can overcome problems of (generally) low computer literacy among older people, as suggested by previous research [38,39].

According to interviewees, future active ZWIP users are interested in managing their own (or their loved one’s) care and experiencing current health problems. Such observations are consistent with major dimensions of the theoretical models of information and communication technology adoption, such as the Unified Theory of Acceptance and Use of Technology model, namely performance expectation, effort expectancy, facilitating conditions, computer experience and voluntariness of use, and social influence [33]. Performance expectancy is defined as the degree to which individuals believe that using the system will help them attain gains in performance. Effort expectancy is the degree of ease associated with the use of the system. Facilitating conditions are defined as the degree to which an individual believes that an organizational and technical infrastructure exists to support use of the system. Computer experience is if patients have used computers before, voluntariness of use is how far patients felt they had a choice in using the system, and social influence is having the feeling that others also use the system and their opinions on the system. Specifically, in terms of performance expectations, patients were more likely to use ZWIP if they expected improved communication with the physician. In terms of effort expectancy, on average they perceived ZWIP use to be easy, although there is a group who find the computer a real barrier to ZWIP use. Patients were also more likely to use ZWIP if they were facilitated in ZWIP usage or had previous computer experience.

Additionally, ZWIP usage was more likely if patients voluntarily participated in ZWIP, and in terms of social influence, actually got messages. Therefore, it seems that the successful use of patient networks requires providers to be willing to respond to patient messages and actively send messages to patients. Conversely, health care providers who decline invitations or fail to respond to patient messages form a serious barrier for implementation. Further research is needed to quantitatively explore the role of technology acceptance factors, for example, using the Unified Theory of Acceptance and Use of Technology model [33] to guide implementation of POHCs in older populations. However, this first study to identify the characteristics of successful ZWIP participants already allows for a stricter selection of patients who might most easily benefit from such interventions. As long as a large group of frail persons experiences the electronic nature of POHCs as a major barrier to use, the efficacy and effectiveness of POHCs can be better tested in more selective samples [40,41]. Carefully selected older people should be more successful in digital self-management.

### Implications for Implementation and Practice

Although we have found some examples of frail older patients in their 80s successfully using ZWIP, low computer literacy of older people seems to hamper the implementation efforts. In the Netherlands, only 39% of older people aged 75 years and older report having Internet access [42], whereas in the younger populations this is close to 100%. One option is to give older people computer training. Previous research has shown that training older people to use computers and health websites that they are expected to use can be successful [43]. Therefore, we have attempted train older people in using computers and the ZWIP website [44]. However, older people were not receptive to our chosen method of implementation of receiving trainers in their homes [44]. An alternative is involving the informal caregivers at an earlier stage, as both our qualitative results and quantitative results revealed the importance of having an informal caregiver for continued use. It is likely that the general level of computer literacy in older people will increase over time. However, low computer literacy will remain problematic in disadvantaged groups or very old people for a considerable time and may be a persistent problem for older people suffering cognitive impairment. In future studies, various implementation methods should be compared to raise computer literacy in the older people and to increase the willingness of informal caregivers to participate. In addition, the effect of using eHealth interventions on various components of health—namely physical, social, and mental health—should continue to be explored further.

### Strengths and Limitations

This study reports a process evaluation of POHC usage in a population of frail older people. Its major strength lies in the mixed methods approach, combining both qualitative and quantitative information on usage, and in its relatively large sample size. Its outcome, a profile of a successful older eHealth user, makes it possible to target likely users. Adequately targeting audiences who will use a new intervention, represents a precondition for studies aiming to show the benefits of such interventions. Thus, these profiles form a valuable contribution to future research and implementation projects of eHealth interventions.

The study also has some limitations to consider. First, this process evaluation of usage was conducted in the Netherlands within a strong primary care system and so it can be generalized only to countries with a similar system. Additionally, we did not continuously measure the health status of individuals, which was shown qualitatively as a main driver of usage, and this should be explored in further research. Finally, while we explored patients’ levels of computer literacy, access to computers, and user-friendliness of ZWIP for the individual older person in the interviews, we omitted a quantitative assessment of these issues and this should be pursued further during evaluation of other applications.

### Conclusions

The current evaluation of use of the ZWIP, a personal online health community for frail older people, revealed important predictors of usage of eHealth interventions in older populations. Frail older patients with poor health status reflected in functional problems, with at least 5-6 providers involved perceived the most benefit and therefore used ZWIP more actively. Sufficient computer skills in either the patient or the informal caregiver and an interest to play an active role in their own care were essential. Therefore, during implementation of POHCs, and probably other eHealth interventions, informal caregivers should be involved from the start of the project. Profiling successful users can facilitate more effective targeting of frail older patients for implementation of eHealth interventions in the near future.

## Multimedia Appendix 1

ZWIP usage.

## Multimedia Appendix 2

Interview questions.

## Abbreviations

ADL: Activities of Daily Living
Easycare-TOS: Two-step Older person Screening
IADL: Instrumental Activities of Daily Living
POHC: Personal Online Health Community
SF-36: Short Form-36 Health Survey
ZWIP: Zorg en Welzijn Informatie Portaal (Health and Welfare portal)
