# Supplementary material for: Which Frail Older Patients Use Online Health Communities and Why? A Mixed Methods Process Evaluation of Use of the Health and Welfare Portal
Source: J Med Internet Res. 2014 Dec 17;16(12):e278. doi: 10.2196/jmir.3609 (PMC4275472; doi:10.2196/jmir.3609)
Supplement: Supplementary file 2 [file jmir_v16i12e278_app2.pdf]

Appendix 2 interview questions

Instructions for interviewers:

As many open questions, and keep asking questions.

What I basically want to find out what you expected to achieve with ZWIP, what is difficult using ZWIP, do they have adequate support, how does their social environment relate to ZWIP. I also want to know if people naturally want to use ZWIP, or just doing the GP a favour.

Questions round one.

1 What exactly is your feeling ZWIP? (cute, scary)

2 How easy or difficult you find ZWIP?

3 What do you think of the possibility to communicate with your caregivers using ZWIP?

4 What do you think of the opportunity to have more influence on your own care through ZWIP?

5. How much support you get from the general practice or all kinds of people using ZWIP?

6 Would you recommend ZWIP to others? Why yes, why not?

Additional round 2:

everyone

1.-Do you use ZWIP? (and who uses ZWIP here actually, the patient or the informal caegiver?

2 Has anyone recommended ZWIP, if yes who?

3. When did you last talk with someone about ZWIP? How was that?

4 What were your expectations ZWIP? What was your reason to join?

For people who used ZWIP often

1 Why do you think ZWIP went as well for you as it did? (Personal characteristics, conditions)?

2 What was the role of the GP (or practice nurse) in ZWIP?

3 Which parts did you use, which parts not, and why?

For people who did not use ZWIP often

1 What hinders you in ZWIP use, and what was the role of the GP in this?

2 What was the role of the GP (or practice nurse) in ZWIP?

3 What had been necessary to use? WHEN would have you used ZWIP?

4 Have you ever really wanted to use ZWIP?

5. Can you cope well with your PC (computer)?
